# Is Dementia Screening of Apparently Healthy Individuals Justified?

**DOI:** 10.1155/2017/9708413

**Published:** 2017-08-08

**Authors:** Larry W. Chambers, Saskia Sivananthan, Carol Brayne

**Affiliations:** ^1^Department of Health Research Methods, Evidence and Impact, McMaster University, Hamilton, ON, Canada; ^2^Bruyère Research Institute, Ottawa, ON, Canada; ^3^Faculty of Health, York University, Toronto, ON, Canada; ^4^Institute for Clinical Evaluative Sciences, Toronto, ON, Canada; ^5^Primary Health Services Division, Alberta Health Services, Edmonton, AB, Canada; ^6^Cambridge Institute of Public Health, School of Clinical Medicine, University of Cambridge, Cambridge, UK

## Abstract

Despite efforts to raise awareness and develop guidelines for care of individuals with dementia, reports of poor detection and inadequate management persist. This has led to a call for more identification of people with dementia, that is, screening individuals who may or may not complain of symptoms of dementia in both acute settings and primary care. The following should be considered before recommending screening for dementia among individuals in the general population.* Dementia Tests*. Low prevalence reduces positive predictive value of tests and screening tests will miss people who have dementia and identify people who do not have dementia in substantial numbers.* Clinical Issues*. The clinical course of dementia has not yet been shown to be amenable to intervention. Misdiagnosis and overdiagnosis can have significant long-term effects including stigmatization, loss of employment, and autonomy.* Economic Issues*. Health systems do not have the capacity to respond to increased demand resulting from screening. In conclusion, at present attention to life-course risk reduction and support in the community for frail and cognitively impaired older adults is a better use of limited healthcare resources than introduction of unevaluated dementia screening programs.

## 1. Introduction

Dementia is a condition with multiple causes that affects memory, other cognitive abilities, and behaviour that interfere with a person's ability to drive, deal with their finances, manage their healthcare, and live independently [1]. Also, dementia has a significant impact not only on individuals but also on their careers, families, communities, and societies [2].

Alzheimer's disease and vascular dementia are the most common forms of dementia and other causes are dementia with Lewy bodies and a group of diseases that contribute to frontotemporal dementia [2]. In 2015, dementia affected 47 million people worldwide, that is estimated to increase to 75 million in 2030 and 132 million by 2050 [3]. Recent reviews estimate that globally nearly 9.9 million people develop dementia each year [3].

Though age is by far the strongest known risk for the onset of the common dementias, not all older adults develop dementia. Also, younger adults develop dementia (defined as the onset of symptoms before the age of 65 years) accounting for less than 9% of cases [2].

Risk reduction and maintenance of cognitive abilities is known to be associated with particular behaviours and environments, similar to other long-term diseases [4]. Dementia risk is lower for people who have been physically active, avoided overweight, had balanced diets, did not smoke, and had moderate use of alcohol as well as avoiding and managing specific conditions such as high blood pressure, stroke, and diabetes mellitus [4].

In our view, there is a profound disconnect between the assertions of some researchers and journals and the wider practitioner and other researcher clinician communities that healthy populations be screened for dementia to identify individuals with dementia early [5–8]. “Early” here includes screening in situations where neither the individual nor his/her caregiver have noticed any symptoms, or have judged, or recognised them, as needing clinical assessment. “Healthy” here refers to the WHO definition, “the extent to which an individual or group is able to realize aspirations and satisfy needs, and to change or cope with the environment. Health is a resource for everyday life, not the objective of living; it is a positive concept, emphasizing social and personal resources, as well as physical capacities” [9].

While the motivation for screening is well intentioned, four recent reports [10–13] by expert panels that systematically reviewed the dementia screening evidence recommend not screening individuals who are not themselves, or their families, seeking such attention (Recent Reviews on Dementia Screening). We think that the following case example can help illustrate different perspectives and assessment of the balance of potential benefit and harm.

*Recent Reviews on Dementia Screening.* See [10–13].

## 2. Case Presentation (Hypothetical)

The local Alzheimer Association requested advice from the local medical society to introduce an awareness raising campaign to screen for dementia. Practitioners often see individuals who once diagnosed have a clear view that their delay in accessing support and advice from services have caused harm and that this can be remedied by systematic screening. They feel that early detection and remediation could lead to improved quality of life for affected individuals and their caregivers. The Alzheimer Association states that their supports and other activities are available for any individuals with identified problems. They believe that making the test results known to caregivers and the Association will assist individuals when identified early. Many jurisdictions have begun such campaigns and are enthusiastic about them. Concerned caregivers in your community are encouraging the Alzheimer Association to follow suit. You are part of a committee asked by the medical society to prepare an answer. Your report will be due next week.

## 3. Use of Critical Appraisal to Assist in Deciding Whether or Not to Screen

Public resources are involved in this decision. Funding used for one activity is not then available for another. Deciding to screen or not should therefore be based on explicit criteria. A collection of previous experiences and the evolution of rules of evidence about screening [14] were fused into the three following areas: testing, clinical decisions, and economic considerations. These criteria can be used to determine, on the basis of current evidence, whether screening causes more harm than good in individuals who have not sought medical attention. The rest of this article will describe these and show how to use them when appropriate for a given population (Table 1).

## 4. Dementia Tests

### 4.1. How Feasible Is It to Accurately Identify Individuals with High Likelihood of Dementia via Screening?

Psychometric properties differ according to populations and settings (community, tertiary, secondary, and primary care). The test quality for dementia is influenced by many factors that include age, culture, occupation, education, environmental context, and health variables (medications, delirium, and depression).

A recent systematic review reported 22 short validated cognitive tests [15], so practitioners have to consider which to use and interpret the results, taking into account the setting and the individual patient's premorbid education, language and literacy skills and any current motor, and hearing and visual impairment. For example, two dementia screening tests used by primary care practitioners for apparently healthy individuals are the Mini Mental State Examination [16] and the Montreal Cognitive Assessment [17]. However, as many as 1 in 8 healthy individuals screened for dementia and mild cognitive impairment are incorrectly classified using the MMSE. As many as 1 in 4 are screened incorrectly using the MoCA [12, 18, 19]. This level of “false positives” can cause harm from an emotional health and practical stand point. In some jurisdictions, a diagnosis for dementia may cause denial of health insurance and may affect an individual's right to drive (see also Section 6.2 below).

Tests for dementia are just the beginning of the process required to arrive at a diagnosis of dementia. Features and typical trajectories of dementia include an understanding of the differences between expected changes and ones that signal deficits related to disability. Test interpretation requires acknowledging the wide variability in changes in cognitive ability over time, in older adults. In addition, medication, including polypharmacy, and comorbid conditions must be taken into consideration. In usual older people the “normal ranges” of cognitive tests have not been tested in whole populations. Screening tests applied in clinics will not perform in the same way in different population settings (see Section 4.2. below).

The diagnosis of dementia must rule out treatable conditions that contribute to cognitive deficits and declines. These include sleep apnea, hypothyroidism, depression, polypharmacy, delirium, and declines in vision and hearing. If a primary care team screen targeted groups who, themselves or their families/community, have not sought help for any concerns and are aged 80 years and over the process will identify many other conditions: high blood pressure (41%), depression (32%), heart disease (27%), transient ischaemic attack (18%), and diabetes (13%) [20].

### 4.2. What Is the Estimated Prevalence of Dementia in the Population to Be Screened?

**Figure 1:**
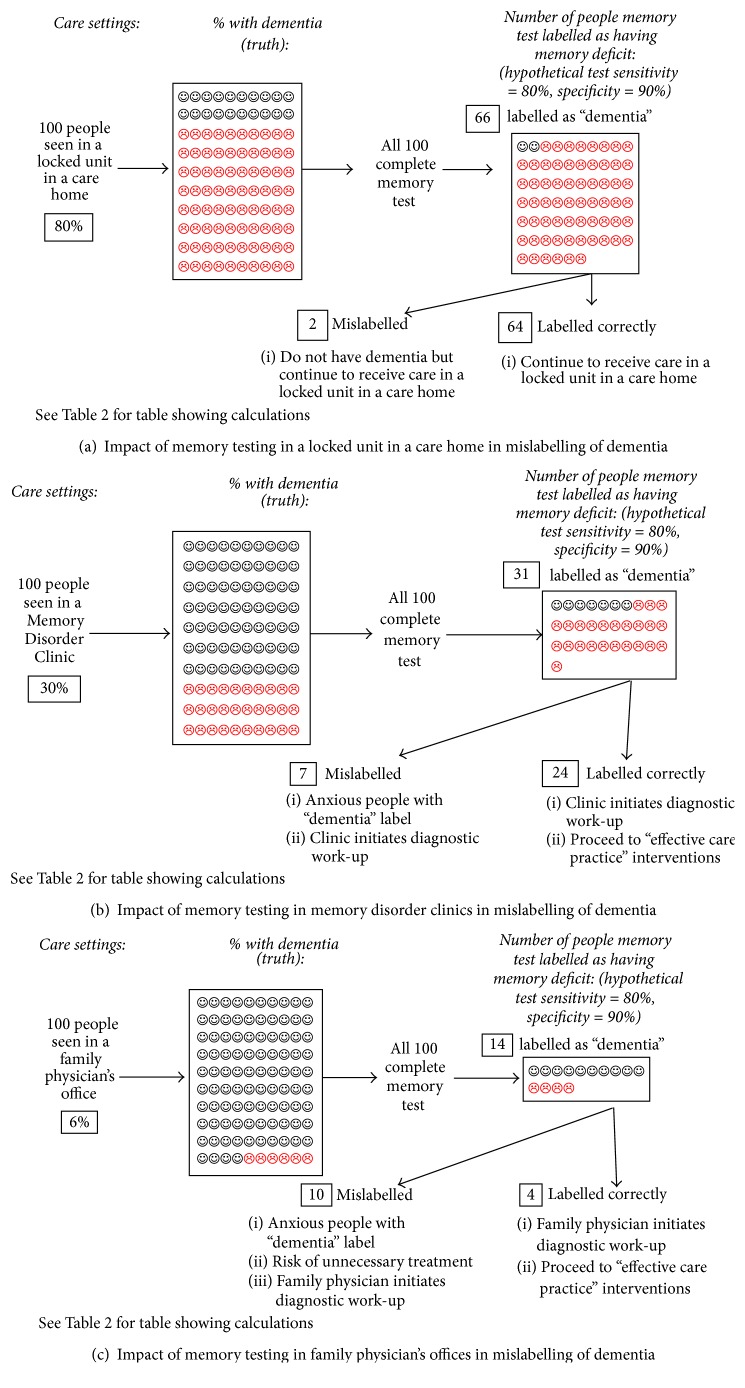


Positive predictive value (PPV) is the probability that a positive test result is correct in those who test positive. The prevalence of dementia in the population being screened has a dramatic impact on the PPV of a test, even if there is consistent sensitivity and specificity. As shown in Figure 1(a) (Table 2), in a locked unit in a long-term care home, where the prevalence of dementia is 80%, regardless of the accuracy of the test, the PPV is 97%. In a Memory Clinic where the prevalence of dementia is 30%, the PPV of the test is 77% (using the same test with equal sensitivity and specificity) (Figure 1(b)) (Table 2). Even in this setting 1/4 people will receive the possibility that their positive screening is wrong. Prevalence of dementia is ~6% [21] in the general population or in a primary care clinic and this results in the PPV of the test dropping to 34% even with the sensitivity and specificity being held steady (Figure 1(c)) (Table 2). This means that between 6 and 7 people out of every 10 who tested positive will not then be diagnosed as demented.

Primary and community care settings involve screening thousands of individuals. Figure 1(c) demonstrates that 10% of individuals screened in these settings will be mislabeled. The number mislabeled would be 100,000 if a million individuals were screened in the general population or in a primary care clinic. However, “… the magnitude of these figures in even an opportunistic screening program result in problems not only at the primary care level but also on the specialist level to handle the volumes of people with a suspected cognitive impairment” [22].

Between 1997 and 2011, incentives in the UK lead to a dramatic increase in the number of referrals, but it led to no increase in the prevalence of dementia [23]. However, in 2014, the United Kingdom Department of Health introduced a £55 incentive for general practice physician offices to screen and diagnose dementia [24]. Memory Disorder Clinics reported having more individuals with functional memory problems and an increase in persons with depression following this policy. Testing of low B12 and folate levels consistently occurred. Mood testing only occurred in 26% of the population being screened for dementia despite knowledge that depression is a common and treatable risk factor for dementia [25]. In 2016, the monetary incentive for general practice physicians in the United Kingdom to screen and diagnose dementia was phased out.

An alternative argument to screening for dementia among apparently healthy older adults has been put forward to screen for general well-being [26]. Here the argument is that the evidence shows that older adults with higher well-being are able to adapt their psychological well-being to numerous impairments and diseases [27, 28]. The resilience and flexibility to adapt are the norm up to very old age, and only in the last period of life, severe disability, or frailty does well-being decline [29]. Using well-being assessment measures in primary care clinics could reveal those individuals who have room for improvement in well-being and they may be the best candidates for cognitive testing. However, “…if an older person or his family has realized a stable and well-adapted way of living, even in the case of cognitive decline, added value of treating for cognitive decline is highly questionable and is best delayed” [26]. In summary, current testing methods are inaccurate, so that not only are people misdiagnosed with dementia, but also true cases are missed.

## 5. Clinical Issues

### 5.1. Is the Clinical Course of Dementia Amenable to Intervention and If Yes at What Stage and Is There Adequate Evidence?

Most common dementias are attributed to a combination of Alzheimer's type and vascular pathologies [30, 31]. The average age of onset of dementia is in a person's early 80s [21]. Mild stages of dementia are being identified increasingly in clinical practice, with a drift toward predementia diagnosis at younger ages.

**Figure 2:**
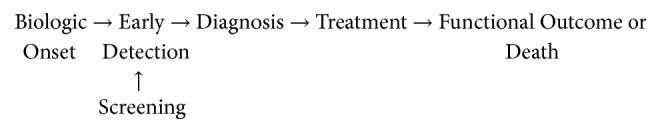
Screening: assumptions about the clinical course of a health condition.

Screening should occur after any condition's biologic onset but before usual diagnosis and treatment to improve the course of consequences of having the condition. The prerequisite assumption is that screening for dementia among previously undiagnosed individuals leads to improvement in quality of life vis-à-vis unscreening individuals (Figure 2).

Although very important for decision-making about interventions, the stages of dementia (mild, moderate, and severe) lack clinical consensus and therefore the stage of dementia is not routinely reported by clinicians. The pathology accompanying dementia among older individuals is complex [32–36]. New research on biomarkers may or may not change this situation.

Screening of apparently healthy individuals is only justified if there is an effective treatment for specific conditions identified. Screening of populations or groups before symptoms develop is clearly not justified at present as there is no drug that arrests clinically diagnosed dementia of the Alzheimer's type. Clinical trials of cholinesterase inhibitors have not been shown to improve the symptoms of dementia with individuals who have mild cognitive impairments [10]. In addition, the substantial number of withdrawals from trials suggests intolerance to these drugs [37]. As many as 40% of individuals assessed as ‘mild cognitive impairment no dementia' may remain the same in the following five years and many improve [38, 39].

### 5.2. What Are the Reasons and Value of an Individual Knowing a Diagnosis of Dementia?

Some argue that screening of apparently healthy individuals is a process that is independent of the evidence of effectiveness of screening but more a “right to know” about the condition. The “right to know” a diagnosis [40] assumes the existence of a precise definition of the condition. An understanding of the condition and the underlying reasons in its clinical course and the clinical course for the person, their age, and gender is also assumed. The assumption continues with the impact of contextual factors such as comorbidity, ethnicity, and education as well as, in the case of dementia, relationship to end of life. Prognostication is one aspect of a diagnosis, but even superficial consideration of the current evidence highlights the lack of a sufficient evidence base to inform such a justification for a diagnosis through screening of apparently healthy individuals [41]. More research is warranted before any further calls for dementia screening of populations.

Prognosis with the dementias and predementia states is already beset with uncertainty, even within a usual clinical setting. Rarely is an individual provided with the scale and range of uncertainty. Where prognosis is being imposed on a previously undiagnosed individual through opportunistic application of a screening test, it would seem important that they are informed of the degree of uncertainty about prognosis of testing positive. This parallels with breast cancer screening where public awareness of overdiagnosis and overtreatment is growing [42, 43].

Screening leads to some misdiagnosis and overdiagnosis for individuals including the harmful effects of diagnosis, implications of taking diagnostic processes further, and overtreatment. The consequences of dementia testing could include being inaccurately labelled, stigmatization, premature curtailment of employment, and loss of support for day-to-day functions, such as driving, remaining independent, and making financial and healthcare decisions [44, 45].

## 6. Economic Issues

### 6.1. Does the Healthcare System Have the Capacity to Respond to Increased Demand Resulting from Screening for Dementia?

Healthcare system challenges required to respond to increased prevalence are likely to be substantial. For example, primary care costs and specialist costs of diagnosing dementia far exceed costs of diagnosing other common chronic conditions for which diagnostic markers are available (e.g., hypertension). This is a small fraction of the lifetime costs of care incurred by an individual with dementia [46].

Dramatic increases in memory clinic referrals following increased screening of “apparently healthy” individuals creates challenges for health and social services [10–13]. Unimpaired and worried individuals may be kept under follow-up which uses up staff resources. This overburdening of existing systems violates one of the principles of screening. Opportunity costs can include fewer resources for those individuals diagnosed with dementia because of underresourced and poorly coordinated health and social care that involves multiple agencies and practitioners [47].

### 6.2. Will Screening Add Value to Individuals with Dementia, the Healthcare System, and Society?

Many members of the public believe that a treatment exists to reduce the existing symptoms and effectively slow its progression. A significant proportion believe that a reliable medical test to diagnose dementia is currently available [48]. The reality is that diagnosis is not always straightforward and there is no drug to stop most causes of dementia [49]. There is a degree of uncertainty that should be shared and understood that the process of diagnosis has a number of uncertainties. This uncertainty is increased because of the connection between cognitive ability, neurobiology, ageing, end of life, and clinical course for an individual. Informed consent is difficult to achieve with this kind of uncertainty.

Recent studies of populations are revealing new concerns about the value of screening to identify early dementia. If referred to a specialist, an individual is twice as likely to be institutionalized [50]. After the first year for people newly diagnosed with dementia, 65% will be transferred from institution to institution, usually acute care hospitals, at least once and 17% will have three or more healthcare practitioner transitions [51]. These transitions can increase the risk of medication errors, hospital readmissions, and deaths. When receiving antipsychotic or benzodiazepine medications or living in a rural area, the transitions increase. Older adults receiving newly started cholinesterase inhibitor drug therapy have more physicians providing care and have higher anticholinergic drug burden scores [52]. These studies raise questions about the importance of the psychosocial environment surrounding the person with dementia. This includes domains of social networks (number of caregivers involved in care decisions) or personality traits (self-care behaviour, being unaware of cognitive inability) that may influence both care referral and the clinical evolution. Overdiagnosis of dementia is harmful and can interfere with a good quality of life because of the altered self-perception and perception of others as well as stigmatization, early institutionalization, side effects of medications, and premature shutdown of regular life activities [42–45].

## 7. Case Conclusion

The medical society group that was advising the local Alzheimer Association about the proposed community wide awareness campaign on dementia screening examined the evidence about the links of “apparently healthy” individuals. It concluded that there were few interventions of rigorously demonstrated efficacy for the symptoms of dementia that would be detected among apparently healthy individuals, and there was no acceptable evidence that the early detection of most of the symptoms of dementia altered their prognoses. The required studies simply had not been done indicating the need for more research on the six questions outlined in this report.

The committee shared the caregivers and the Alzheimer Association concerns that a substantial burden of morbidity affecting individuals, their caregivers, and the whole community resulted from dementia.

The measurement properties of the available and feasible screening tests were largely unknown in primary care settings.

Moreover, it was judged that the oldest, old individuals who were at the greatest risk for dementia, would be most likely the group that might benefit from dementia screening. The committee questioned whether dementia screening of apparently healthy individuals was the best use of existing Alzheimer Association and other specialized services.

The committee's final recommendation was that the resources available for the community-wide awareness campaign on dementia screening should be used for research to generate evidence on dementia screening in the community.

## Figures and Tables

**Table 1:** Six testing, clinical, and economic conditions for determining whether screening for dementia among “apparently” healthy individuals does more good than harm.

*Dementia tests*
(i) How feasible is it to accurately identify individuals with high likelihood of dementia via screening tests?
(ii) What is the estimated prevalence of dementia in the population to be screened?
*Clinical issues*
(i) Is the clinical course of dementia amenable to intervention and if yes at what stage and is their adequate evidence?
(ii) What are the reasons and value of an individual knowing a diagnosis of dementia?
*Economic issues*
(i) Does the healthcare system have the capacity to respond to increased demand resulting from screening for dementia?
(ii) Will screening add value to individuals with dementia, the healthcare system, and society?

**Table 2:** The effect of prevalence on the predictive value of a screening test.

	Prevalence								
	Low (6%) screening^*∗*^			Moderate (30%) case-finding^*∗∗*^			High (80%) diagnosis^*∗∗∗*^		
Test	Definitive diagnosis of condition								
	Present	Absent	Total	Present	Absent	Total	Present	Absent	Total

Abnormal	48	94	142	240	70	310	640	20	660
Normal	12	846	858	80	630	690	160	180	340
Total	60	940	1000	300	700	1000	800	200	1000

	Predictive value of an abnormal test								

	$\frac{48}{142}\times 100=34\%$			$\frac{240}{310}\times 100=77\%$			$\frac{640}{666}\times 100=97\%$		

Hypothetical sensitivity = 80%, specificity = 90%									

^*∗*^All people on a family health roster.  ^*∗∗*^All people in a memory disorder clinic.  ^*∗∗∗*^All people in a locked unit in a long-term care facility.

## Additional Points

Considerable investment has been made by many societies to raise awareness of dementia, including exhortations for screening by some. Four recent reports by expert panels that systematically reviewed the evidence about screening for dementia and recommend not screening individuals who are not themselves, or their families, seeking such attention. Current evidence on testing, clinical decisions, and economic considerations can be evaluated to estimate whether screening could cause more harm than good in individuals who have not presented for medical attention and may have no symptoms. Considerable resources are currently being used for dementia screening without an evidence base and should be redirected and restructured to generate evidence on dementia screening in the community and selected settings.
